# Hospitals and Poor Law Reform

**Published:** 1910-03-26

**Authors:** 


### The Hospital

A Journal of

The Medical Sciences and Hospital Administration.

New Series. No. 162, Vol. VI. [No. 1234, Vol. XLVII.] SATURDAY, MARCH 26, 1910.

### Hospitals and Poor Law Reform.

The interesting address delivered by Sir Henry Burdett last week on the occasion of the annual meeting of the Hospitals Association and published elsewhere in this issue, draws attention to the intimate relation between the reform proposals, embodied in the Reports of the Commission on the Poor Laws, and the future of hospitals and institutions for the relief of the sick poor in this country. The connection between the voluntary hospital and the Poor-law infirmary is doubtless obscure to many, and to superficial observers probably never appears to be so close as it really is. Yet it is an undeniable fact that of late years the distinction on which Sir Henry Burdett laid stress, namely, that the infirmary relieves sick paupers, while the function of the voluntary hospital is primarily to prevent the poorer classes being reduced, through sickness or accident, to the level of absolute poverty, has scarcely been so sharply apparent as it was, say, thirty years ago. The tendency has been in modern times towards pauperising the community, towards creating invidious class distinctions, towards undermining the independence of the wage-earner by exhorting him to put his trust in members of Parliament and looking to the State to secure him those comforts which his forefathers attempted to attain through thrift and self-help. In these circumstances it is only natural that the hospital worker should be gravely concerned about the future of charitable institutions, and should consider very seriously the probable effects of whatever may tend to change and alter existing conditions.

Among the factors that promise to be most potent in affecting such alterations are the reports of the Commission on the Poor Laws issued last year. The proposals regarding medical relief contained in the Majority Report are eminently unsatisfactory. They have not placated the advanced reformers, and they have scarcely commended themselves to thinking men, and the probability is that we shall hear very little about them in the future. The proposals embodied in the Minority Report, on the other hand, have aroused widespread interest, not only because of the thoroughness with which they attack the whole problem, but largely also because of their probable revolutionary character. The lively satisfaction felt by those who are in whole-hearted agreement with the Commissioners responsible for the Minority Report, has been opposed by the vehement antagonism of objectors to these proposals, and the fight is still being waged with more zeal than ever. About the pros and cons of the argument, every practitioner and everyone interested in institutional work should be clear, and for that reason we recommend readers to study the Minority Report for themselves, and to make themselves acquainted with the objections put forward against it.

That these objections are very serious—so grave indeed that they cannot be lightly considered, but must be weighed with most scrupulous care and attention is very evident. As Sir Henry Burdett justly showed, and as most objectors have pointed out, acceptance of the proposals put forward by the minority involves the creation of a State medical service, the expenditure of a huge sum of money annually, and the serious probability of the break-up of the present voluntary hospital system. Whether these results are desirable or not, we need not for the moment stop to argue, nor whether they are inevitable or preventible. The main fact is that they are possibilities, and as such should be very carefully considered. The financial position of the hospitals under the voluntary system, and thanks to the success which has attended the creation of the central fund, is improving every day. If balance sheets could ever have justified that system, it is justified now. But while this is a highly satisfactory state of affairs, so far as hospitals are concerned, it is permissible to ask how long is it going to last if additional burdens are to be put upon the community, if a huge competing combine, receiving preferential treatment from the State, is to be created to deal with the indigent sick, and if the already Sisyphean task of the ratepayer is to be doubled by making him bear the expense of treatment of the sick belonging to the wage-earning classes? We venture to think that in those circumstances the inevitable result must be that the voluntary system will break down. People will certainly not subscribe to an institution for the relief of the sick poor when they are already supporting a State medical service; and, in fact, the existence of a voluntary system of hospital relief alongside a State system of such magnitude as the Minority Report proposes is a logical absurdity. If the State service is efficient the *raison d'être* of the voluntary hospital, not supported by the rates, is gone; the latter then sinks to the level of a mere private undertaking with private aims and a limited scope. On the other hand, if the voluntary hospitals are efficient, if they are worked upon a scheme of co-operation such as has been suggested, helped by the State and municipalities, and helping these in their turn, not dependent upon these nor controlled by them, but acting as self-dependent units in a great combination, their usefulness would be much increased, their position strengthened, and their future assured.

There is here an opportunity, as Sir Henry Burdett has said, for an enthusiastic statesman, with knowledge of his subject, with business experience, with sympathy and a capacity for taking unlimited pains, to do the nation a signal service by elaborating a scheme of co-operation between hospitals conducted on the voluntary system, infirmaries under the Poor Law, and medical relief work under the State. There is scope, too, for modification of the proposals of the Minority Report in consonance with such a co-operative scheme, and we sincerely trust that those who endorsed these proposals will not deem it too late in the day earnestly to consider such modifications. Meanwhile we look to the medical profession and to all those interested in institutional work to bestir themselves in the consideration and discussion of these proposals and their probable effects. It is only by such full and unfettered discussion that conclusions which are worth notice can be arrived at, and the whole subject, which so vitally touches the profession and the hospitals alike, adequately ventilated.

